# ATR-FTIR spectroscopy reveals genomic loci regulating the tissue response in high fat diet fed BXD recombinant inbred mouse strains

**DOI:** 10.1186/1471-2164-14-386

**Published:** 2013-06-10

**Authors:** Ayca Dogan, Peter Lasch, Christina Neuschl, Marion K Millrose, Rudi Alberts, Klaus Schughart, Dieter Naumann, Gudrun A Brockmann

**Affiliations:** 1 Department for Crop and Animal Sciences, Humboldt-Universität zu Berlin, Invalidenstraße 42, 10115, Berlin, Germany; 2 Robert Koch-Institute, Biomedical Spectroscopy, Nordufer 20, 13353, Berlin, Germany; 3 Department of Infection Genetics, Helmholtz Centre for Infection Research and University of Veterinary Medicine Hannover and University of Tennessee Health Science Center, 38124, Braunschweig, Germany

**Keywords:** Collagen, Endoplasmic reticulum, Apoptosis, Remodeling, Liver steatosis, Viperin, Collectin-11

## Abstract

**Background:**

Obesity-associated organ-specific pathological states can be ensued from the dysregulation of the functions of the adipose tissues, liver and muscle. However, the influence of genetic differences underlying gross-compositional differences in these tissues is largely unknown. In the present study, the analytical method of ATR-FTIR spectroscopy has been combined with a genetic approach to identify genetic differences responsible for phenotypic alterations in adipose, liver and muscle tissues.

**Results:**

Mice from 29 BXD recombinant inbred mouse strains were put on high fat diet and gross-compositional changes in adipose, liver and muscle tissues were measured by ATR-FTIR spectroscopy. The analysis of genotype-phenotype correlations revealed significant quantitative trait loci (QTL) on chromosome 12 for the content of fat and collagen, collagen integrity, and the lipid to protein ratio in adipose tissue and on chromosome 17 for lipid to protein ratio in liver. Using gene expression and sequence information, we suggest *Rsad2* (viperin) and *Colec11* (collectin-11) on chromosome 12 as potential quantitative trait candidate genes. *Rsad2* may act as a modulator of lipid droplet contents and lipid biosynthesis; *Colec11* might play a role in apoptopic cell clearance and maintenance of adipose tissue. An increased level of *Rsad2* transcripts in adipose tissue of DBA/2J compared to C57BL/6J mice suggests a cis-acting genetic variant leading to differential gene activation.

**Conclusion:**

The results demonstrate that the analytical method of ATR-FTIR spectroscopy effectively contributed to decompose the macromolecular composition of tissues that accumulate fat and to link this information with genetic determinants. The candidate genes in the QTL regions may contribute to obesity-related diseases in humans, in particular if the results can be verified in a bigger BXD cohort.

## Background

During the development of obesity, the many changes in adipose tissue eventually lead to the disruptions of its normal dynamic endocrine function. Besides its storage ability of excessive lipids, the adipose tissue produces and secretes many factors that are involved in the pathophysiology of obesity associated secondary diseases like diabetes, dyslipidemia, liver steatosis, hypertension and cardiovascular disease [1,2]. Obesity-associated organ-specific pathological states can be ensued from the dysregulation of circulating levels of adipokines and metabolites and ectopic fat storage within organs that normally do not accumulate fat, e.g. liver and muscle. Excessive fat accumulation is accompanied by changes in tissue composition, architecture, and tissue remodeling [3-5]. How these processes affect the function of tissues is not well understood.

It is known that individual differences in the development of adiposity are the result of genetic predisposition and environmental factors. Several studies in mice have identified quantitative trait loci (QTLs) that influence various obesity related traits. For example, QTLs have been identified for regional adiposity [6], diet induced obesity [7], resistance to diet induced obesity [8], juvenile obesity [9], obesity associated diseases [10,11] as well as lipid content in the liver [12].

The dissection of fat depots [6], dual energy X-ray absorptiometry (DEXA) [13,14], or magnetic resonance imaging (MRI) [9,13] have been used to measure fat pad size and body fat distribution. Chromatographic techniques (gas chromatography, HPLC) [13], different biochemical reagent sets [8], or enzymatic assays [10] are used for metabolic profiling to obtain information about specific components such as cholesterol, triglycerides, glycerol and others. Most of these methods are time consuming and not suitable for liquid and solid samples without preparation. Furthermore, they cannot specify structural and compositional changes in the samples, simultaneously.

Attenuated total reflectance (ATR) spectroscopy represents a well-established sampling method in infrared (IR) spectroscopy [15]. In ATR-FTIR spectroscopy an IR beam is typically guided through an IR transparent crystal (typically ZnSe or Ge) in a way that one (or several) total internal reflections take place at the inner surface of the crystal. This creates an evanescent or near-field standing wave at the boundary between the crystal and the surrounding media (air). If the samples are prepared directly on the surface of the ATR crystal their absorption properties can be observed as a result of attenuation of the evanescent wave. The penetration depth of the radiation is typically at the order of the wavelength and depends on factors like the wavelength, the angle of incidence and of the refractive indices of the crystal material and the sample. An ATR spectrum thus carries only information of a thin sample layer close to the ATR crystal. The ATR approach allows measurements of samples like blood, serum, or fully hydrated tissues samples, without too much interference from IR absorption of bulk water [16]. Drawbacks of the ATR technique are possible molecular interactions between the crystal surface and the sample.

The IR method is based on the characteristic absorption of infrared radiation at specific wavelengths by functional groups like N–H, C=O, C–H and P=O [17]. An IR spectrum carries specific information on the samples’ molecular composition and structure (protein, lipid, carbohydrate and nucleic acids etc.). As each sample represents a highly characteristic combination of molecules, IR spectroscopy provides a phenotypic fingerprint that is complementary to genomic approaches to detect unique genetic variants between individuals. Its high sensitivity allows the detection of differences among subspecies or strains [18]. One of the main advantages of the IR technique is speed. Results can be obtained within a few minutes. Furthermore, only a small amount of material with a minimal or no sample preparation is required to apply this method [18,19]. Therefore, ATR-FTIR spectroscopy can be considered as a high-throughput technique that is very practical for studies with high sample numbers as it is the case in genetic mapping experiments. It is an automated, nondestructive, sensitive and reproducible technique, which is easy to perform [20]. Because of its many advantages, the IR spectroscopy has become established as a research tool in biomedical applications for body fluids [21,22], pathological changes in tissues [23-25], diagnosis of diseases [26,27], and cell line classification and discrimination, in particular in cancerous tissue [20,28].

In the present study, we applied the Attenuated Total Reflectance Fourier Transform Infrared Spectroscopy (ATR-FTIR) to obtain quantitative information about macromolecular composition in adipose tissue as major fat storage depot, and in liver and muscle, which may store ectopic fat. The lipid to protein ratio and collagen integrity were also assessed to identify molecular and compositional changes within distinct tissues. In the obese state, the expansion of adipose tissue is associated with an increased collagen content, which contributes to tissue stiffening and fibrosis [29]. As remodeling of the tissue occurs, the alterations in the environment of the collagen amide bonds may cause changes in the collagen molecule structure. Unraveling of collagen triple helix can occur and be monitored from the spectral changes such as a shift in the absorbance frequency [30]. The collagen band at 1338 cm^-1^ is sensitive to the ordered structure of the triple helix. Reduction of the intensity of this band indicates collagen denaturation [31]. Therefore, changes in the area of this band account for changes in the ratio of areas of the amide II band at 1550 cm^-1^ to the collagen band at 1338 cm^-1^, which we refer to as collagen integrity [30].

The goal of this study was to identify genes that regulate obesity in BXD recombinant inbred (RI) strains of mice [32]. The association of the genomic mosaic structure of the genomes of every RI strain with the phenotypic differences between strains allows the mapping of genomic regions accounting for the trait variance. The BXD RI strains descend from crosses between C57BL/6J (B6) and DBA/2J (D2) mice and subsequent inbreeding by repeated brother sister mating over more than 20 generations [32]. They have been used to identify genetic factors contributing to health complications such as atherosclerosis [33], blood pressure [34], diabetes [35] and obesity [36,37]. The parental strains C57BL/6J and DBA/2J, which were originally crossed to generate the set of BXD RI strains, also differ in their susceptibility to high fat diet induced obesity [38] and regional fat storage patterns [39]. Combining the sensitive ATR-FTIR study with the well characterized genetic model, we found differences in the macromolecular composition of adipose and liver tissues among high fat diet-fed BXD RI strains, which led to the identification of new genomic regions that may control obesity-related diseases.

## Results

### Phenotypes of parental and BXD RI strains on high fat diet

**Figure 1:**
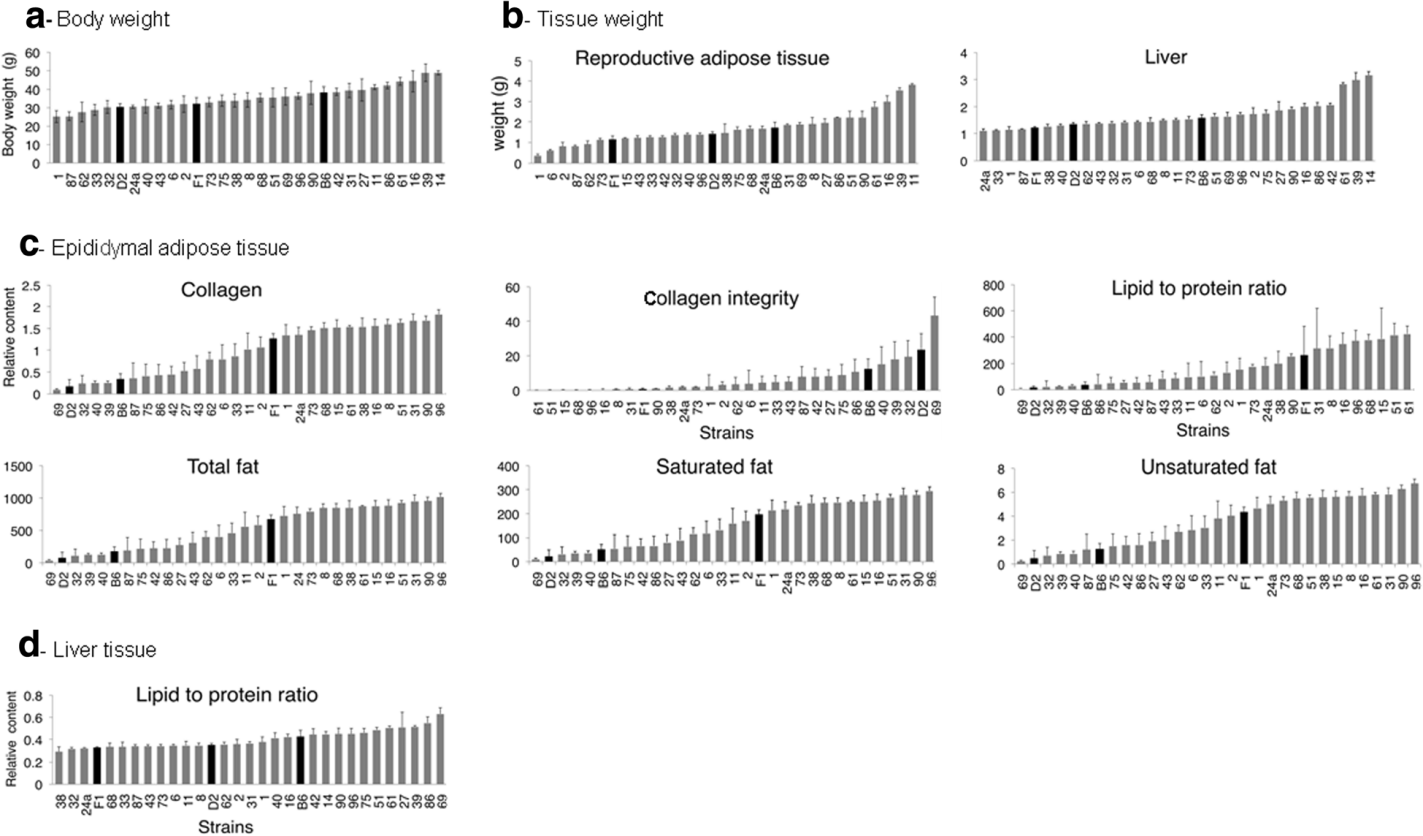
**Rank ordered strain distribution patterns for different traits in 29 BXD RI strains. a**. Body weight. **b**. Weights of epididymal adipose tissue and liver. **c**. Content of total, saturated and unsaturated fat, collagen content, collagen integrity and the ratio of lipid to protein content in epididymal adipose tissue. **d**. Ratio of lipid to protein content in the liver. Data represent means and standard errors of four to five 20 weeks old males per strain after feeding a high fat diet over 16 weeks. FTIR-data are given as relative units as described in Experimental Procedures. Black columns mark the parental strains, B6 and D2, and F1 animals. The distribution patterns are only shown for traits in which significant QTLs were found.

After feeding a high fat diet over 16 weeks, B6 males gained more weight compared to D2 males (Figure 1a). They had more than two-fold higher relative contents of total fat, saturated fat, unsaturated fat, collagen and lipid to protein ratio (Table 1) and 0.5 fold lower relative collagen integrity in the adipose tissue. Similar results were found in the liver. In contrast, muscles of B6 mice had a higher relative collagen integrity than D2 mice.

**Table 1:** Comparison of compositional spectral parameters for target tissues in the parental strains B6 and D2

		**Total fat content**	**Saturated fat content**	**Unsaturated fat content**	**Acyl chain length**	**Collagen content**	**Collagen integrity**	**Lipid to protein ratio**	**Glycogen content**
Epididymal adipose tissue	B6	178.70 ± 68.98	51.47 ± 20.59	1.23 ± 0.49	20.66 ± 0.62	0.35 ± 0.12	12.49 ± 5.79	37.80 ± 1.41	_
	D2	77.74 ± 87.57	22.00 ± 26.77	0.49 ± 0.64	19.67 ± 0.64	0.17 ± 0.16	23.59 ± 9.30	17.99 ± 3.01	_
Liver	B6	2.75 ± 0.57	1.25 ± 0.34	_	11.02 ± 1.79	0.90 ± 0.09	712.68 ± 79.60	0.43 ± 0.40	2.13 ± 0.17
	D2	2.20 ± 0.12	0.95 ± 0.06	_	9.62 ± 0.31	0.69 ± 0.03	906.20 ± 44.88	0.35 ± 0.12	1.92 ± 0.21
Muscle	B6	1.48 ± 0.97	0.81 ± 0.55	_	9.88 ± 2.08	7.44 ± 0.01	78.91 ± 15.26	0.25 ± 2.29	0.71 ± 0.03
	D2	1.80 ± 0.42	1.02 ± 0.26	_	12.45 ± 1.30	7.64 ± 0.01	77.10 ± 16.15	0.30 ± 0.76	0.73 ± 0.04

The value of each trait content was derived from spectral parameters for comparison and does not refer to the mass.

Therefore, the unit is an arbitrary unit.

Among the 29 BXD RI strains, the intra-strain variation was smaller than the inter-strain variation for all traits (Figure 1) indicating genetic heritability. The 29 BXD RI strains also showed a large diversity for epididymal adipose tissue (0.37±0.08 to 3.82±0.07 g) and liver weights (1.11±0.08 to 3.17±0.13 g) and they differed widely in the macromolecular composition of epididymal adipose tissues and liver (Figure 1). Differences between strains were not significant in muscle.

Many BXD RI strains exceeded the parental values for total, saturated and unsaturated fat contents, collagen content and lipid to protein ratio in adipose tissue and were closer to the F_1_ animals (which exhibited higher values than the high parental strain). Thus, the more extreme phenotypes of many BXD RI strains reflect novel combinations of additive and dominant allele effects in the BXD RI strains as well as novel epistatic interactions between the parental alleles [40].

Pearson’s correlation coefficients were estimated using strains means to evaluate the degree of relationship between traits in the population of all BXD RI strains (Table 2). High correlations (either positive or negative) between traits imply that certain genes or genomic region could simultaneously influence these traits. In our study, the weights of the epididymal adipose tissue and liver (r=0.625, p<0.001) as well as liver and muscle (r=0.521, p<0.01) were highly correlated. For the macromolecular contents of all tissues, significant positive correlations were found between fat related traits. Negative correlations were observed between collagen and collagen integrity in all tissues examined. In the epididymal adipose tissue, collagen integrity showed significant negative correlations with all traits except for the ratio of saturated to unsaturated fat (Table 2). Collagen itself correlated positively with all other traits, except collagen integrity. In the liver, no significant correlation was found between collagen and fat related traits. In muscle, negative correlations of collagen with collagen integrity was found whereas glycogen was positively correlated with acyl chain length, and collagen integrity (Table 2). Between tissues, significant correlations were found for collagen content and integrity between liver and muscle and between unsaturated fat content in epididymal adipose tissue and glycogen content in muscle.

**Table 2:** Pearson`s correlation coefficients between traits in reproductive adipose tissue, liver and muscle of BXD RI strains

	**Traits**	**1**	**2**	**3**	**4**	**5**	**6**	**7**	**8**	**9**	**10**	**11**	**12**	**13**	**14**	**15**	**16**	**17**	**18**	**19**	**20**	**21**
Epididymal adipose tissue	1: total fat (15084)																					
	2: saturated fat (15083)	0.998 ***																				
	3: unsaturated fat (15082)	0.998 ***	0.998 ***																			
	4: saturated to unsaturated fat ratio (15086)	0.436	0.438	0.384																		
	5: acyl chain length (15085)	0.894 ***	0.897 ***	0.893 ***	0.306																	
	6: collagen (15089)	0.999 ***	0.999 ***	0.998 ***	0.425	0.898 ***																
	7: collagen integrity (15090)	−0.885 ***	−0.886 ***	−0.893 ***	−0.231	−0.91 ***	−0.893 ***															
	8: lipid to protein ratio (15087)	0.911 ***	0.908 ***	0.899 ***	0.489	0.804 ***	0.903 ***	−0.784 ***														
Liver	9: total fat (15092)	−0.201	−0.2	−0.21	−0.083	−0.18	−0.218	0.291	−0.054													
	10: saturated fat (15091)	−0.221	−0.22	−0.23	−0.091	−0.175	−0.238	0.289	−0.075	0.986 ***												
	11: acyl chain length (15093)	−0.203	−0.202	−0.215	−0.039	−0.148	−0.219	0.325	−0.076	0.873 ***	0.822 ***											
	12: collagen (15096)	0.316	0.309	0.326	−0.089	0.333	0.309	−0.343	0.279	0.07	0.001	0.105										
	13: collagen integrity (15097)	−0.275	−0.268	−0.289	0.148	−0.273	−0.268	0.315	−0.265	−0.154	−0.083	−0.17	−0.965 ***									
	14: lipid to protein ratio (15094)	−0.226	−0.226	−0.232	−0.126	−0.226	−0.244	0.323	−0.063	0.985 ***	0.965 ***	0.873 ***	0.05	−0.165								
	15: glycogen (15098)	−0.107	−0.109	−0.1	−0.139	−0.083	−0.112	0.06	−0.045	0.286	0.24	0.42	0.228	−0.378	0.371							
Muscle	16: total fat (15100)	0.198	0.199	0.188	0.072	0.231	0.191	−0.152	0.208	0.417	0.419	0.387	−0.122	0.144	0.378	0.05						
	17: saturated fat (15099)	0.211	0.212	0.201	0.078	0.246	0.204	−0.168	0.22	0.426	0.43	0.393	−0.109	0.131	0.386	0.045	0.998 ***					
	18: acyl chain length (15101)	0.323	0.323	0.317	0.113	0.324	0.314	−0.168	0.31	0.312	0.248	0.505	0.093	−0.072	0.293	0.161	0.732 ***	0.729 ***				
	19: collagen (15104)	−0.066	−0.059	−0.08	0.189	−0.021	−0.058	0.157	−0.067	0.098	0.165	−0.06	−0.582	0.633 *	0.047	−0.251	0.456	0.438	0.175			
	20: collagen integrity (15105)	0.179	0.172	0.19	−0.132	0.173	0.169	−0.24	0.172	−0.073	−0.146	0.094	0.629 *	−0.649 **	−0.036	0.21	−0.273	−0.253	0.016	−0.928 ***		
	21: lipid to protein ratio (15102)	0.149	0.151	0.137	0.095	0.168	0.144	−0.097	0.167	0.415	0.416	0.379	−0.202	0.221	0.378	0.042	0.989 ***	0.985 ***	0.698 ***	0.517	−0.365	
	22: glycogen (15106)	0.573	0.568	0.582 **	0.061	0.527	0.562	−0.489	0.537	0.095	−0.013	0.323	0.55	−0.549	0.108	0.227	0.216	0.225	0.615 *	−0.478	0.649 **	0.126

The degree of significance was denoted as: p<0.05*, p<0.01**, p<0.001***, p values were adjusted for multiple testing.

GeneNetwork Record ID was given in parentheses for each trait.

Regardless of the tissue, the estimated heritability was higher than 0.60 for the content of fat, collagen and glycogen.

### QTL analysis

Linkage analyses provided evidence for two genomic regions contributing significantly to differences in the macromolecular composition of epididymal adipose tissue and liver.

**Figure 2:**
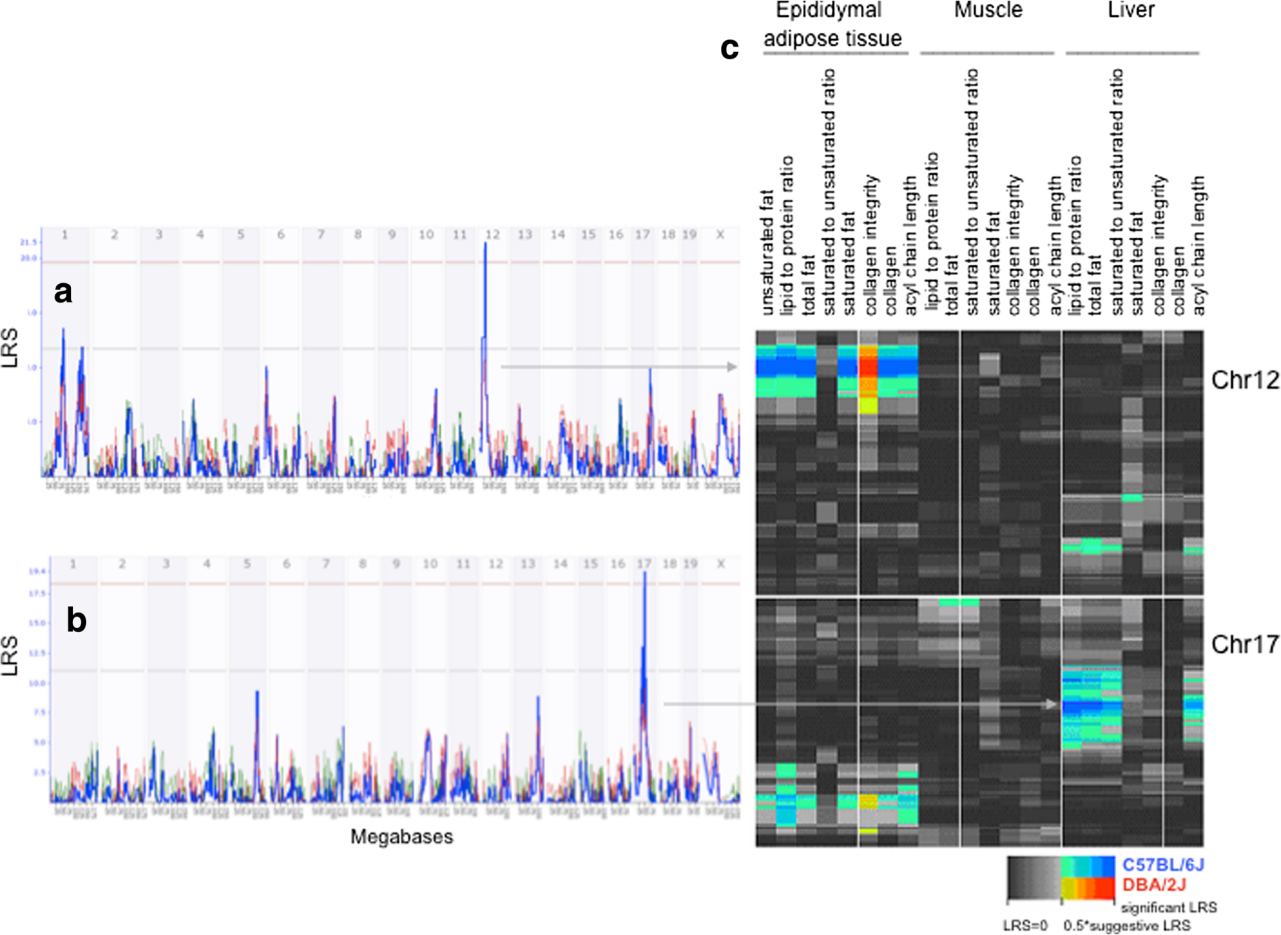
**Genome wide linkage analyses in BXD RI strains.** The QTL scan was performed for all traits, but we illustrate only the trait with the most significant LRS scores for each set of traits that were mapped to the same QTL interval. **a**. QTL scan for total fat content in adipose tissue (GeneNetwork Record ID 15084). A significant QTL was mapped on Chr 12. This QTL region had also effects on the contents of saturated and unsaturated fat, acyl chain length, collagen content and integrity, and the ratio of lipid to protein. **b**. QTL scan for the ratio of lipid to protein in the liver (GeneNetwork Record ID 15094). A significant QTL was mapped on Chr 17. This QTL region was overlapping with the contents of total and saturated fat and acyl chain length. The numbers on the top of figures represent chromosomes. The y-axis and the bold blue function provide the LRS (LRS=4.6 x LOD) values. The two horizontal dashed lines indicate the genome-wide significant (upper line) and suggestive thresholds (lower line) at p<0.05 and p<0.63, respectively. The thin red and green lines indicate the average additive effects of D2 (green) and B6 (red) alleles at particular markers. **c**. The heat map shows QTL regions for different traits obtained by ATR-FTIR spectroscopy. Overlapping QTL regions were detected on chromosomes 12 and 17 in epididymal adipose tissue, liver, and muscle. Intense colors show chromosomal regions with high linkage statistics (LRS) and the spectrum encodes the allelic effect. These figures are directly taken from GeneNetwork [62].

**Figure 3:**
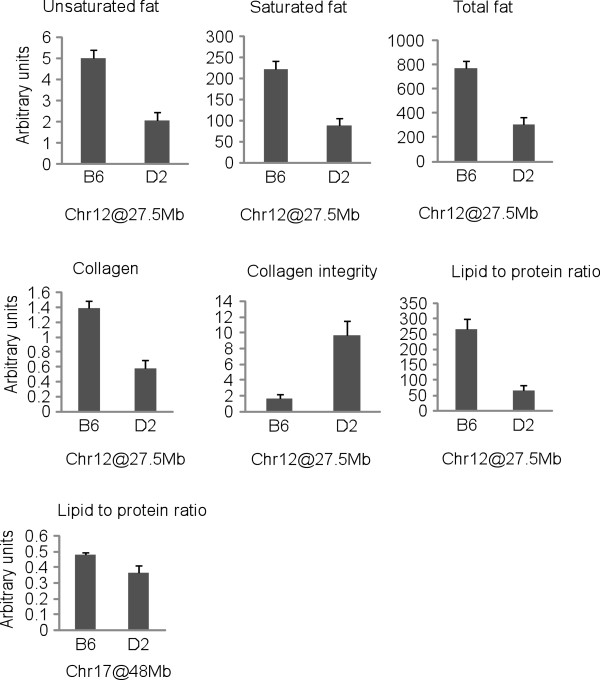
**Genotype effects on affected traits of QTL positions on Chr 12 (rs13481357) and 17 (rs13483011).** Values are means ± SE. B6 and D2 are homozygous for the C57BL/6J and DBA/2J alleles, respectively.

A genomic region on chromosome (Chr) 12 between 26 and 30 Mb significantly affected the relative contents of total, saturated, and unsaturated fat, as well as the relative content of collagen, collagen integrity and lipid to protein ratio in adipose tissue (Figure 2 and Table 3). The effect on the length of the acyl chain was suggestive. The B6 allele of the Chr 12 locus was the increasing allele for all traits, except for collagen integrity. The pleiotropic effect of this locus is consistent with the high correlation between these traits (Table 2). BXD strains being homozygous for the B6 allele at the Chr 12 QTL had about 2.5 fold more relative contents of total, saturated and unsaturated fats, and collagen content and 3.9 fold higher lipid to protein ratio than BXD strains being homozygous for the D2 allele. The collagen integrity was 5.5 fold lower in homozygous B6 than D2 carriers (Figure 3).

**Table 3:** QTLs for the macromolecular composition of different tissues in BXD RI strains

**Tissue**	**Chr**	**Trait**	**LRS**	**Significance**	**Increasing allele**	**Additive Effect**
Reproductive	1	total fat	13.61	*	B6	198.54
Adipose		(15084)	11.92	*	B6	188.92
Tissue	1	saturated fat	13.71	*	B6	57.78
		(15083)	12.07	*	B6	55.09
	1	unsaturated fat	13.4	*	B6	1.26
		(15082)	11.76	*	B6	1.20
	1	acyl chain length	13.16	*	B6	0.36
		(15085)	10.82	*	B6	0.36
	1	collagen	13.24	*	B6	0.34
		(15089)	11.95	*	B6	0.33
	1	collagen integrity	10.62	*	D2	3.39
		(15090)	11.96	*	D2	3.80
			11.38	*	D2	3.49
	9	saturated to unsaturated ratio (15086)	10.58	*	B6	1.05
	12	total fat (15084)	21.47	**	B6	236.62
	12	saturated fat (15083)	21.47	**	B6	68.65
	12	unsaturated fat (15082)	21.24	**	B6	1.51
	12	acyl chain length (15085)	16.27	*	B6	0.40
	12	collagen (15089)	22.09	**	B6	0.42
	12	collagen integrity (15090)	17.65	**	D2	4.16
	12	lipid to protein ratio (15087)	20.95	**	B6	99.76
	17	lipid to protein ratio	14.27	*	B6	86.64
		(15087)	13.69	*	B6	85.26
Liver	5	total fat (15092)	12.49	*	B6	0.30
	5	saturated fat (15091)	11.73	*	B6	0.15
	9	collagen (15096)	14.48	*	B6	0.10
			12.31	*	B6	0.10
	9	collagen integrity (15097)	13.28	*	D2	76.10
			11.69	*	D2	73.89
	17	total fat (15092)	16.72	*	B6	0.35
	17	saturated fat (15091)	13.84	*	B6	0.17
	17	acyl chain length (15093)	12.55	*	B6	1.17
	17	lipid to protein ratio	13.04	*	B6	0.05
		(15094)	19.36	**	B6	0.06
	18	glycogen (15098)	10.9	*	D2	0.22
Muscle	5	collagen integrity (15105)	11.72	*	B6	20.77
	9	collagen (15104)	17.33	*	D2	1.03
	9	collagen integrity	17.19	*	B6	23.45
		15105)	16.81	*	B6	23.60
	9	Glycogen (15106)	12.31	*	B6	0.27
	15	collagen (15104)	11.1	*	D2	0.86

The degree of LRS significance was denoted as: suggestive*, significant**. Genome-wide significance (p<0.05) and suggestive (p<0.63) thresholds were calculated as likelihood ratio statistic (LRS) in linkage analyses computed for 1000 permutations [60]. The values of total, unsaturated and saturated fat, the ratio of saturated to unsaturated fat, collagen and collagen integrity, the ratio of lipid to protein and glycogen are relative values as described in experimental procedures. GeneNetwork Record ID was given in parenthesis for each trait.

The 4 Mb QTL region on Chr 12 affecting the composition of adipose tissue is sparse of genes and harbours only 24 protein coding genes (Additional file 1: Table S1). The genes encoding the radical S-adenosyl methionine domain containing 2 (*Rsad2*) and collectin sub-family member 11 (*Colec11*) matched with the criteria for potential quantitative trait candidate genes, e.g. location of the gene in the QTL region, expression in the target tissue, density of non-synonymous (ns) SNP and InDels, cis-regulation of expression, and known or presumed gene function.

Two nsSNPs between the parental strains B6 and D2 are located in exon 1 of *Rsad2*. The SNP rs29136357 with the alternative alleles G and C in the strains B6 and D2, respectively, leads to an amino acid exchange from Aspartate (Asp) to Glutamate (Glu) at the amino acid position 57 (ENSMUSP00000020970). The SNP rs13467134 with the alleles G and A in B6 and D2, respectively, causes an amino acid exchange from Proline (Pro) to Leucine (Leu) at position 55 (ENSMUSP00000020970). In the *Colec11* gene, the SNP rs29221025 was found as non-synonymous polymorphism in exon 1 with the alleles C and G in B6 and D2, respectively. This SNP causes an amino acid change from Valine (Val) to Leucine (Leu) at the amino acid position 147 (ENSMUSP00000049285). So far, no functional consequences were reported for those changes in the protein structures of RSAD2 and COLEC11.

Expression data of *Rsad2* and *Colec11* in adipose tissues of B6 and D2 on a standard diet are accessible at BioGps [41,42]. Both genes are expressed in adipose tissue. According to those data and normalisation of expression levels with housekeeping genes (*Actc, Gapdh, Rps29, B2m, Ppia, Gusb, Tbp*) *Rsad2* and *Colec11* transcript amounts are 2.78 and 1.93 times higher in B6 than in D2 mice, respectively, under standard diet conditions [42] when D2 animals are heavier than B6 animals.

**Figure 4:**
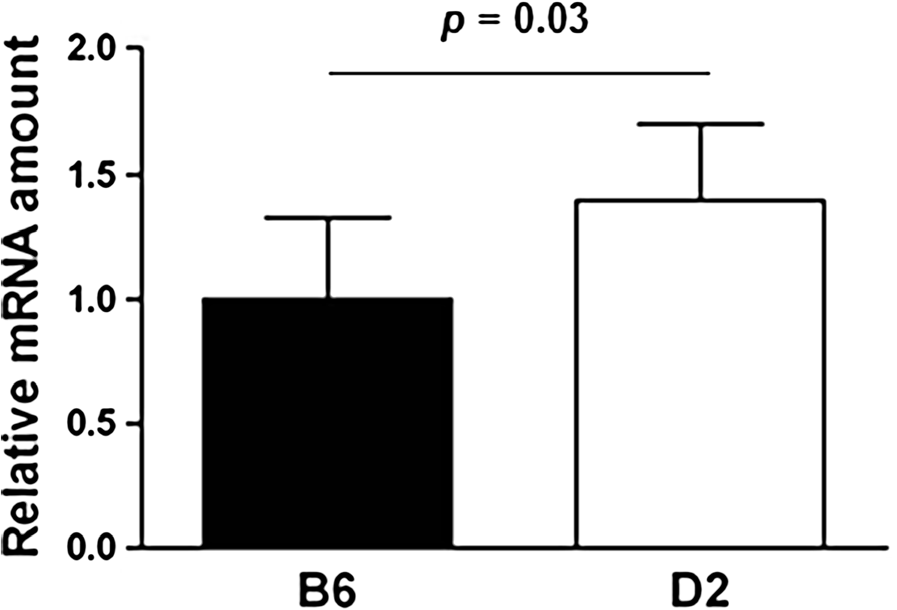
**Relative mRNA expression of 20 weeks old males on high fat diet.** Relative mRNA expression level of *Rsad2* from epididymal adipose tissue. Different BXD RI strains carrying either the C57BL/6J (B6) or DBA/2J (D2) allele at the position of the target gene were chosen randomly from the BXD recombinant inbred strains (one animal per strain) for gene expression analyses. Bar graphs with different letters are significantly different to a level of significance of p = 0.03; n = 6–8. Statistics were performed using the two-tailed Student’s t-test and bar graphs are mean values plus SD.

Evidence for cis-regulation comes from genetic variation between B6 and D2 and expression QTLs (eQTL) for *Rsad2* and *Colec11* in segregating F_2_-populations. For example, for both genes, eQTLs were described for adipose tissues in the cross CastXC57BL6/J (CastB6/B6Cast F_2_), and in addition for *Colec11* in the cross C3H/JxC57BL6/J (BH/HB F_2_) (GeneNetwork references: Probeset 10018174238 for *Rsad2* in adipose tissue of cross CastB6/B6CAST F2, Database: UCLA CTB6/B6CTF2 Adipose (2005) mlratio; Probeset 10024397101 for *Colec11* in adipose tissue of cross CastB6/B6CAST F2, Database: UCLA CTB6/B6CTF2 Adipose (2005) mlratio; Probeset 10024397101 for *Colec11* in adipose tissue of cross BH/HBF2, Database: UCLA BHHBF2 Adipose (2005) mlratio). Because four out of 10 synonymous SNPs in the coding region of *Rsad2* that exist between B6 and D2 also occur between Cast/J and B6 mice, it is very likely that the genetic variation in this gene could be responsible for expression or functional differences in both reference populations. The measurement of transcript amounts of *Rsad2* and *Colec11* in our high fat diet fed BXD RI strains carrying the alternative B6 and D2 alleles of the respective genes revealed 1.4 times higher mRNA amounts of *Rsad2* of D2 carriers (p=0.03), while no expression difference was found for *Colec11* (p=0.10) (Figure 4). These results suggest cis-acting genetic variation that interacts with environmental changes on the cellular level leading to differential gene activation of *Rsad2* under different diets.

A second significant QTL region affecting the lipid to protein ratio in the liver was found on Chr 17 in an interval between 46 and 50 Mb. The same region influenced the relative content of total and saturated fat and acyl chain length. For all traits, the B6 allele was the allele associated with an increase in phenotype value. This gene-rich region harbours 100 protein coding genes (Additional file 2: Table S2), among them genes contributing to apolipoprotein production, mitochondrial function, and tissue structure. Because of the high gene number, it is presently not possible to perform a search for candidate quantitative trait genes.

In respect to glycogen content, no significant effect was found in adipose tissues. Suggestive genomic regions affecting glycogen content in the liver and in the muscle were detected on chromosomes 18 and 9, respectively (Table 3).

## Discussion

In the present study, a phenotypic characterization method, ATR-FTIR spectroscopy, was used to characterize structure and composition of adipose, muscle and liver tissue in 29 BXD RI mouse strains. The main purpose of this study was to combine IR data with genetic information in order to identify specific gene loci responsible for the observed differences in the macromolecular tissue composition. The sensitive tissue characterization and high quality of data obtained by ATR-FTIR technique allowed us to identify novel chromosomal regions contributing to distinct features of high fat diet induced obesity. Furthermore, the specific haplotype block structure of the set of BXD RI strains allows the mapping of trait associated effects to specific chromosomal regions: a 4 Mb interval on Chr 12 correlated with relative fat content in epididymal adipose tissue, and a 4 Mb on Chr 17 correlated with lipid to protein ratio in the liver.

We did not find significant QTLs for the muscle phenotypes. One reason for missing effects may be the lack of variance between the parental strains B6 and D2. Another reason may be the preparation method of the muscle tissue for ATR-FTIR measurements, because homogeneity is crucial in the IR spectroscopy to obtain accurate data from the tissue. Therefore, homogenous tissues like adipose and liver tissues give robust and reliable data. In contrast, the quadriceps muscle is composed of four different muscle groups (*Musculus rectus femoris*, *Musculus vastus intermedius*, *Musculus vastus lateralis*, and *Musculus vastus medialis*), which may differ in the macromolecular composition. Here, we used the whole sliver of the muscle. This is likely the reason, why possible differences between mouse groups could not be detected.

The identified QTLs on Chr 12 and 17 in our study coincided with previously mapped QTLs for adipose tissue, body and liver weights in the LGXSM advanced intercross line (AIL) [43]. But, the confidence intervals in our study are much smaller. The small genomic QTL regions are the result of a high recombination rate during the generation of RI strains. Moreover, the whole genome sequence information of both parental strains B6 and D2 allows for the selection of candidate genes that could cause the phenotypic differences.

The mapping of highly correlated phenotypes such as the relative content of total, saturated and unsaturated fat and lipid to protein ratio to the same narrow chromosomal region on Chr 12 suggests pleiotropic gene effects. In other words, it is likely that a functional relationship exists between these correlated traits. To nominate genes that might underlay the identified QTLs, six filters were applied including location, expression in the target tissue, density of nsSNP and InDels, cis regulation of expression, and function of gene. This approach and additional gene expression analyses which we performed with BXD RI strains on a HFD provided further data to suggest *Rsad2* on Chr12 as a positional candidate gene affecting fat content variation in epididymal adipose tissue and the lipid to protein ratio in the liver, respectively. Higher transcript amounts of *Rsad2* transcripts were always found in the tissues with the lower fat deposition. On SBD, D2 mice had leaner livers than B6 and on a HFD B6 was leaner than D2. The observation of higher gene expression in the leaner tissue is in line with cis-acting genetic variation interacting with environmental factors that occur in different amounts in the cells, e.g. fatty acids.

*Rsad2* (aliases; viperin, *vig1, cig5*) is an evolutionary conserved protein. Initially, it was identified as an antiviral protein of innate immunity to diverse pathogens [44]. *Rsad2* is located on the cytosolic side of the endoplasmic reticulum (ER) membrane through an N-terminal amphipathic α-helical domain. This domain contains a leucine zipper motif, which is involved in protein-protein interactions and may affect the proper folding of the protein as well as anchoring the protein to the ER [44,45]. Recently, it has also been shown that *Rsad2* co-localizes with the adipocyte differentiation-related protein (ADRP), which is located in the outer phospholipid and protein layer of lipid droplets and interacts with farnesyl–diphosphate synthase, an enzyme, which is located on the ER membrane and which is required for the generation of cholesterol [46,47]. The overexpression of *Rsad2* inhibits the secretion of various soluble proteins, induces changes in the ER morphology [45]. The ability to change the ER membrane could also affect the lipid droplet formation and morphology. Furthermore, *Rsad2* may also alter the lipid content and quantity in lipid droplets, which originate in the ER, by binding farnesyl–diphosphate synthase [46]. Our gene expression data for *Rsad2* supports findings of other expression experiments showing that increased bodily fat content is associated with lower *Rsad2* expression [48]. The co-localization of *Rsad2* on lipid droplets as lipid storage organelles, the association of lower gene expression with higher fat deposition and our genetic mapping results suggests that *Rsad2* controls the formation of lipid droplets while impairment of *Rsad2* likely enhances fat accumulation in lipid droplets. It has been repeatedly demonstrated that obesity and obesity-induced inflammation can arise from ER stress, by the accumulation of unfolded or misfolded proteins in the ER lumen and by overburden the reserve capacity of the organelle to tolerate it [49]. It has been shown that *Rsad2* is likely also required for the T-cell receptor mediated activation of *NFkB* and *AP-1*, which are important regulators of inflammatory cytokine production in white adipose tissue (61,62). Given the functional properties of *Rsad2* for lipid biosynthesis and proper protein folding and ER stress, *Rsad2* might affect the activation of inflammation in an obese status.

*Colec11* belongs to the C-type lectins of the collectin family that is composed of a collagen-like domain and a carbohydrate recognition domain [50]. Recently, *Colec11* has been identified as a member of the innate immune system. The collectins have functions in inflammatory and allergic responses, apoptotic cell recognition and clearance, and in the adaptive immune system [51]. In particular the function of apoptotic cell recognition and clearance of *Colec11* could contribute to the growth and maintenance of white adipose tissue by adipose tissue regeneration. Although we did not find gene expression differences of *Colec11* (Chr12) mRNA amounts, we cannot exclude protein quality changes that might affect the function of COLEC11 and thus the collagen content in epididymal adipose tissue.

The suggested candidate genes *Rsad2* and *Colec11* may also contribute to human diseases as mutations leading to deleterious functional changes in the proteins have been identified in humans (Additional file 3: Table S3) [52].

With regard to the QTL for lipid to protein ratio on Chr17, further fine mapping is necessary to reduce the number of positional candidate genes. Because this QTL resides in a gene-rich genomic region, many potential candidate genes were predicted. Among the 100 protein coding genes in the QTL interval there are several that might affect the lipid content and the lipid to protein ratio in the liver. For fine mapping additional BXD strains could be phenotypically characterized, which harbor recombinations in the QTL confidence interval.

## Conclusion

In the present study, we introduced the analytical technique of ATR-FTIR spectroscopy as a novel phenotyping method that allows to characterize the macromolecular composition of different tissues and that minimizes the measuring error arising from non-molecular methods. Using this sensitive method, we found differences among the BXD RI strains with respect to the trait of interest, which reflect genetic variation. The analyses revealed two genomic regions that may have a function in obesity-induced tissue dsyfunction. As candidate gene on Chr 12 we suggest *Rsad2,* which may modulate lipid droplet contents and lipid biosynthesis, and *Colec11,* which may play a role in apoptotic cell clearance and maintenance of adipose tissue. Further functional studies, for example with transgenic or knockout models, are required to validate these candidate genes. For the QTL on Chr17, fine mapping is necessary to reduce the number of candidate genes. However, even if the QTL results are significant, they were identified in a small population of just 29 BXD strains. Therefore, a replication study with a bigger panel of BXD strains would be necessary to verify the findings.

Our study represents a first and crucial step to identify genetic causes of alterations in the tissue specific macromolecular composition in the obese state. These results will help to gain detailed insight into individual and tissue-specific responses to high fat diet as a function of macromolecular composition and genetic factors that are important in the pathogenicity of obesity and obesity-associated clinical complications. Furthermore, our results showed the suitability and efficiency of IR spectroscopy to detect component specific changes since modes of vibrations of each group are dependent on changes in chemical composition, structures, conformation and environment. Therefore, combination of analytical techniques such as mass and vibrational spectroscopy with genetic approaches are suggested to help in identifying tissues with altered physiology, increase the power of QTL analysis and support a better understanding of underlying mechanisms.

## Methods

### Animals, housing and diets

A total of 152 males of the parental strains C57BL/6J (B6) and DBA/2J (D2), F_1_ offspring of the initial cross between B6 and D2 (B6D2F1), and 29 BXD RI strains (BXD 1, 2, 6, 8, 11, 14,15,16, 24a, 27, 31, 32, 33, 38, 39, 40, 42, 43, 51, 61, 62, 68, 69, 73, 75, 86, 87, 90, 96) were used in this study (four to five males of each of the parental, F_1_ and BXD strains were used). The BXD RI strains were derived by inbreeding from a B6XD2 F2 intercross in the two subsets. BXD 1 through BXD 42 were generated by Taylor et al. [53] and BXD 43 through BXD 100 (named “William’s strains”) were generated by Peirce et al. [32]. No phenotypic differences were observed between the two subset of BXD RI strains.

Mice were purchased from The Jackson Laboratory (Bar Harbor, Maine, USA, or from The Oak Ridge National Laboratory, Oak Ridge, Tennessee, USA) and were bred in the facility of the Neuro-Bsik consortium from the VU University Amsterdam, Netherlands. At the age of four weeks, mice were shipped to the mouse facility of the Department for Crop and Animal Sciences, Faculty of Agriculture and Horticulture at Humboldt-Universität zu Berlin, Germany.

Mice were maintained under conventional conditions and controlled lighting with a 12:12 hours light:dark cycle at a temperature of 22 ± 2°C and a relative humidity of 65%. They were reared in groups of three to four individuals of the same sex in macrolon cages with a 350 cm^2^ floor space (E. Becker & Co (Ebeco) GmbH, Castrop-Rauxel, Germany) and with bedding type S 80/150, dust-free (Rettenmeier Holding AG, Wilburgstetten, Germany). All individuals had *ad libitum* access to food and water. The animals were treated in accordance to and all experimental protocols were approved by the German Animal Welfare Authorities (approval no: G0182/07).

Beginning at the age of 4 weeks, mice were fed a high-fat diet (HFD) (Ssniff® diet S8074-E010, Germany) until 20 weeks. The diet contained 20.7% crude protein, 25.1% crude fat, 5.0% crude fiber, 5.9% crude ash, 39.7% N-free extract, 20.0% starch, 17.5% sugar, vitamins, trace elements, amino acids, and minerals (19.1 MJ/kg metabolizable energy; thereof 45% energy from fat, 31% from carbohydrates, and 24% from proteins). The fat in the diet derived from coconut oil and suet.

*Tissue Sampling-* Four to five males of each of the parental, F_1_ and BXD strains were used for ATR-FTIR measurements. At 20 weeks, mice were fasted for two hours, anesthetized under isofluorane and decapitated using surgical scissors. After exsanguinations, epididymal fat pads (which were the epididymal adipose tissue), liver, and quadriceps muscle (comprised of *Musculus rectus femoris*, *Musculus vastus intermedius*, *Musculus vastus lateralis*, and *Musculus vastus medialis*) were dissected and weighed. All tissues were immediately frozen in liquid nitrogen and stored at −80°C until ATR-FTIR studies.

### ATR-FTIR measurement

Tissue samples were mounted on a ZnSe ATR crystal of a multisample holder with one internal reflection. The multisample holder was placed into a Bruker IFS 28/B FTIR spectrometer (Bruker Optik GmbH, Ettlingen, Germany). The sample compartment was continuously purged with dry air to minimize spectral contributions from water vapor and carbon dioxide. The IR spectra were recorded at a physical resolution of 4 cm^-1^ in the 600–4000 cm^-1^ region at room temperature. Each interferogram was collected by co-adding 32 scans. A zero filling factor of 4 was employed, yielding a spectral point spacing of 1 cm^-1^.

The penetration depth of IR light in the sample for ATR measurements is independent of sample thickness. Consequently, in ATR-FTIR spectroscopy the spectra are collected from a thin surface layer of the sample. In order to characterize the core of the tissue instead of the superficial layer, samples were cut and the cut surface was brought into close contact with the ZnSe ATR crystal of a multisample holder. Thus, we acquired ATR spectra from the inner parts of organs of interest. Each tissue block was measured three times at the same day but at different position of the multisample ATR crystal. For every tissue sample, a mean ATR spectrum was obtained. These mean spectra were further averaged to yield strain and organ specific ATR-FTIR reference spectra. For each mouse strain, tissues from four to five males were characterized.

### Data preprocessing

First, water vapor correction of the raw spectral data was carried out by using an automatic water vapor correction routine developed in-house [54]. Then, a spectral quality test was applied to all 1368 ATR-FTIR spectra. This test included tests for defined global intensity thresholds, residual water vapor bands and the signal-to-noise ratio. Spectra with a positive quality test result were min-max normalized. This method scales spectrum intensities by setting the minimum absorbance unit to 0 and the maximum to 1. The min-max normalization was carried out in the region between 1700 and 1500 cm^-1^. Spectral parameters obtained from min-max normalized ATR-FTIR spectra were used as inputs for subsequent QTL analysis.

Beside the min-max normalization, we tested also parameters derived from raw and second derivatives spectra. Since differently pre-processed data provided highly similar QTL analysis results we present here only results of the min-max normalization.

### Extraction of spectral features

**Figure 5:**
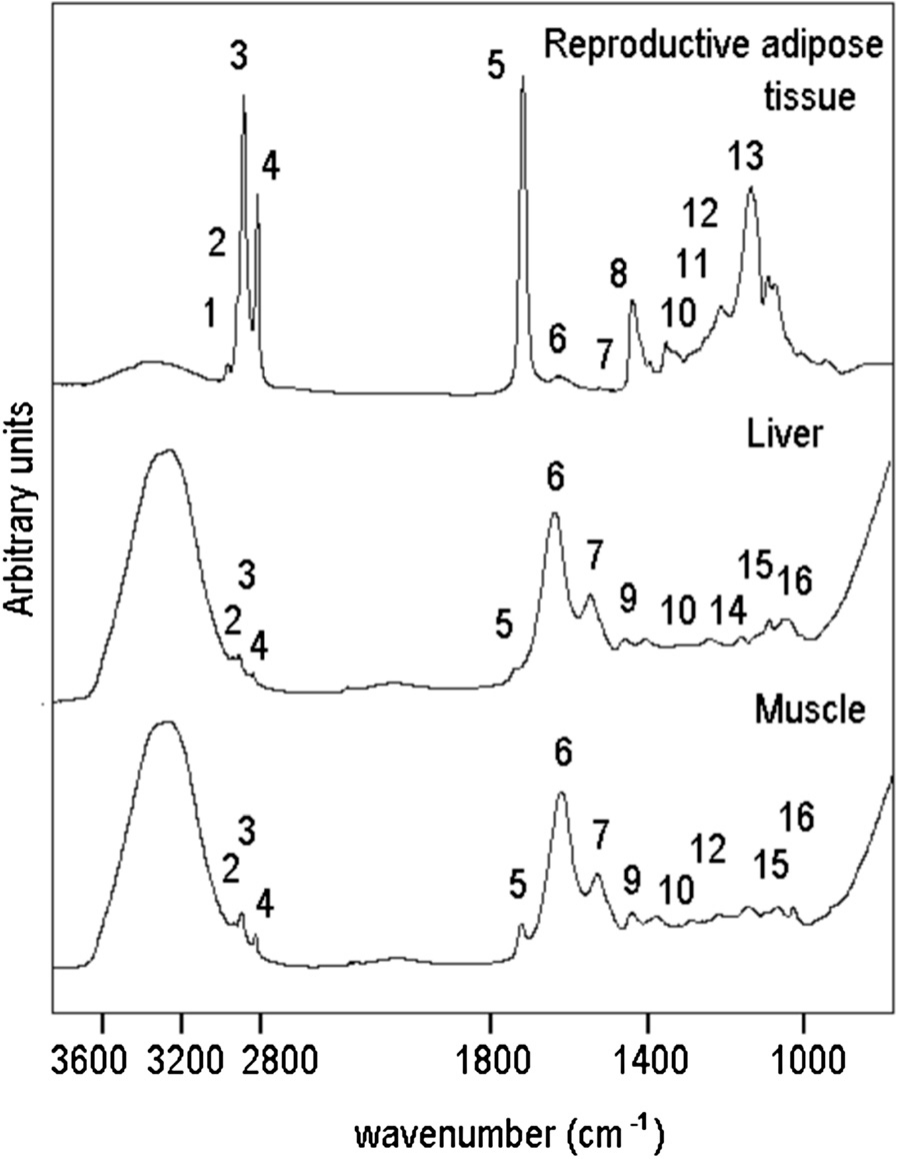
**Representative ATR-FTIR spectrum of epididymal adipose tissue, liver and muscle.** The spectrum was recorded in tissues of a 20 weeks old B6 male mice after feeding a high fat diet over 16 weeks. Numbers refer to peak assignment, which is given in Table 1.

A selection of spectral features obtained from pre-processed ATR-FTIR spectra served as input for the QTL analyses. Most of the spectral features were calculated as integrated intensities, or ratios thereof, according to a method implemented as integration method B in the data acquisition software package OPUS from Bruker Optics (Bruker Optics, Rheinstetten, Germany). Tables 4 and 5 and Figure 5 provide an overview of the positions of the IR bands used and give also the precise frequencies of the integration borders. Note that the precise band positions may be different in different types of tissues.

**Table 4:** **General band assignments in FTIR spectra of reproductive adipose tissue, liver and muscle tissue **[55]

**Peak No**	**Wavenumber (cm**^**-1**^**)**			**Definition of the spectral assignment**
	**Epididymal adipose tissue**	**Liver tissue**	**Muscle tissue**	
1	3006	_	_	Olefinic=CH stretching vibration: lipid (mainly unsaturated)
2	2957	2963	2961	CH_3_ asymmetric stretch: mainly lipid with the little contribution from proteins, carbohydrates, nucleic acids
3	2922	2927	2927	CH_2_ asymmetric stretch: mainly lipid with the little contribution from proteins, carbohydrates, nucleic acids
4	2853	2854	2854	CH_2_ symmetric stretch: mainly lipid with the little contribution from proteins, carbohydrates, nucleic acids
5	1743	1745	1741	Ester C=O stretch: triglicerides, cholesterol esters
6	1646	1638	1638	Amide I (protein C=O stretch)
7	1550	1547	1550	Amide II (protein N–H bend, C–N stretch)
8	1464	_	_	CH_2_ bending vibration: lipids
9	_	1455	1457	CH_2_ bending vibration: lipids
10	1340	1340	1340	CH_2_ side chain vibrations of collagen
11	1320	_	_	collagen
12	1280	_	1282	collagen
13	1160	_	_	stretching vibration of the C-O bond of glycerol skeleton of triglycerides
14	_	1154	_	C-O stretch glycogen
15	_	1081	1081	C-O stretch: glycogen
16	1047	1044	1046	C-O stretching band coupled with C-O bending of C-OH groups of glycogen

**Table 5:** Overview of IR spectral parameters

		**Band area limits**	
**Traits**	**Epididymal adipose tissue**	**Liver tissue**	**Muscle tissue**
A_FAT_	A_3006_+A_2957_+A_2922_+A_2853_+A_1743_+A_1464_+A_1160_	A_2963_+A_2927_+A_2854_+A_1745_+A_1455_	A_2961_+A_2927_+A_2854_+A_1741_+A_1457_
	A_3006_ : A [2997:3017] cm^-1^	-	-
	A_2957_ : A [2950:2966] cm^-1^	A_2963_ : A [2952:2968] cm^-1^	A_2961_ : A [2951:2968] cm^-1^
	A_2922_ : A [ 2905:2936] cm^-1^	A_2927_ : A [2900:2938] cm^-1^	A_2927_ : A [2900:2939] cm^-1^
	A_2853_ : A [2840:2861] cm-^1^	A_2854_ : A [2840:2861] cm^-1^	A_2854_ : A [2840:2861] cm^-1^
	A_1743_ : A [1730:1760] cm^-1^	A_1745_ : A [1737:1759] cm^-1^	A_1741_ : A [1735:1755] cm^-1^
	A_1464_ : A [1454:1474] cm^-1^	A_1455_: A [1437:1475] cm^-1^	A_1457_ : A [1450:1472] cm^-1^
	A_1160_ : A [1133:1200] cm^-1^	-	-
A_SATF_	A_2922_+A_2853_	A_2927_+A_2854_	A_2927_+A_2854_
	A_2922_ : A [2905:2936] cm^-1^	A_2927_ : A [2900:2938] cm^-1^	A_2927_ : A [2900:2939] cm^-1^
	A_2853_ : A [2840:2861] cm^-1^	A_2854_ : A [2840:2861] cm^-1^	A_2854_ : A [ 2840:2861] cm^-1^
A_UNSATF_	A_3006_ : A [2997:3017] cm^-1^	_	_
A_COL_	A_1340_+A_1320_+A_1280_	A_1340_	A_1340_+A_1282_
	A_1340_ : A [1336:1345] cm^-1^	A_1340_: A [1332:1346] cm-1	A_1340_ : A [1334:1355] cm^-1^
	A_1320_ : A [1315:1327] cm^-1^	-	-
	A_1280_ : A [1275:1285] cm^-1^	-	A_1282_ : A [1275:1287] cm^-1^
A_PROT_	A_1550_+A_1340_+A_1320_+A_1280_	A_1547_+A_1340_	A_1550_+A_1340_+A_1282_
	A_1550_ : A [1506:1571] cm^-1^	A_1547_ : A [1500:1565] cm-1	A_1550_ : A [1500:1570] cm^-1^
	A_1340_ : A [1336:1345] cm^-1^	A_1340_ : A [1332:1346] cm-1	A_1340_ : A [1334:1355] cm^-1^
	A_1320_ : A [1315:1327] cm^-1^	-	-
	A_1280_ : A [1275:1285] cm^-1^	-	A_1282_ : A [1275:1287] cm^-1^
A_GLYC_	-	A_1154_+A_1081_+A_1044_	A_1081_+A_1046_
	-	A_1154_ : A [1141:1174] cm-1	-
	-	A_1081_ : A [1071:1091] cm-1	A_1081_ : A [1067:1100] cm^-1^
	-	A_1044_ : A [1020:1050] cm-1	A_1046_: A [1030:1052] cm^-1^
		Band Area Ratio	
saturated to unsaturated fat ratio	(A_2922_+A_2853_)/A_3006_	_	_
acyl chain length	A_2922_/A_2957_	A_2927_/A_2963_	A_2927_/A_2961_
collagen integrity	A_1550_/(A_1340_+A_1320_+A_1280_)	A_1547_/A_1340_	A_1550_/(A1_340_+A_1282_)
lipid to protein ratio	(A_3006_+A_2957_+A_2922_+A_2853_+A_1743_+A_1464_+A_1160_)/ (A_1550_+A_1340_+A_1320_+A_1280_)	A_2963_+A_2927_+A_2854_+A_1745_+A_1455_/ A_1547_+A_1340_	A_2961_+A_2927_+A_2854_+A_174_1+A_1457_/ A_1550_+A_1340_+A_1282_

A_FAT_, total fat; A_SATF_, saturated fat ; A_UNSATF_, unsaturated fat; A_COL_, collagen; A_PROT_, total protein; A_GLYC_, glycogen. “A” is the area obtained by “method B” integration bands in OPUS. The min-max normalized data were used for area calculation.

These parameters were used for the quantitative evaluation of compositional differences in reproductive adipose tissue, liver and muscle of BXD RI strains.

The degree of relationship between traits was estimated on the basis of strain means by Pearson´s correlation coefficients in the population of all BXD RI strains. The p values for significance of the correlation coefficient were adjusted for multiple correlation analysis.

### Quality check of phenotypic data

Analyzing spectral data of all individuals and tissues across all BXD RI strains, we detected outliers having three standard deviations higher or lower than the mean for some traits. To reduce the effect of these extreme values in the interval mapping and correlation analyses, we performed winsorization to bring the upper and lower outliers closer to the nearest non-outlier value of the corresponding distribution for the trait of interest [56]. ATR-FTIR measurements of the epididymal adipose tissue in BXD 14 and of the liver in BXD 15 were detected as outliers and excluded from further analysis. The data is available on GeneNetwork [57] as strain means of each min-max normalized trait. The GeneNetwork identifiers for the traits analysed in this study are given in Tables 2 and 3.

### Heritability

The broad sense heritability was calculated based on variances in the parental strains, F_1_ and BXD RI strains according to Wright [58].

### QTL mapping

For mapping genomic regions contributing to the differences in tissue composition, we used strain means of all phenotypic data of epididymal adipose tissue, liver and muscle and genotypes of a set of 3795 markers of the 29 BXD RI strains [59]. All traits that we measured were of quantitative nature. QTL mapping was carried out by interval mapping using marker regression on GeneNetwork’s WebQTL module [57]. Genome-wide significance (p<0.05) and suggestive (p<0.63) thresholds were calculated as likelihood ratio statistic (LRS) in linkage analyses computed for 1000 permutations [60]. Confidence intervals were defined as 1.5 LOD drop-off (6.9 LRS units) from the peak marker position.

### Candidate gene discovery

GeneNetwork is a platform that contains a database, which stores a collection of phenotypes and gene expression data sets of the BXD RI strains. Furthermore, it combines experimental data and bioinformatics approaches of different experiments to facilitate the identification of candidate genes [61]. Initially, we selected the genes, which are physically located in the target QTL interval with the highest test statistic. Subsequently, we checked the expression of these positional candidate genes in the tissues of interest. We used an expression data set of liver of BXD RI strains, which was provided by GeneNetwork [62] and expression data of adipose tissue of B6 and D2 that was accessible through BioGps [41,42]. Because both strains B6 and D2 were sequenced, we picked sequence variants between B6 and D2 in a region of 10 kb 5´ of the first exon and 1 kb 3´of the last exon of candidate genes and functionally annotated all variants using Candi SNPer [63]. Subsequently, we looked for the density of nonsynonymous SNPs and insertions and deletions (InDels) within candidate genes, which are present between B6 and D2 [57]. After we detected nonsynonymous SNPs or InDels, the potential functional change in the protein was investigated using the programs POLYPHEN [64] and SIFT [65]. Genes harbouring mutations that affect protein sequences or isoform type, genes that harbour variants that affect the mRNA level or both protein variant and transcript amounts are considered as candidate genes. Which candidate genes were associated with cis-regulation was determined with eQTL data from different data sets on WebQTL (BH F_2_ (Apo null), BH/HB F_2_ and CastB6/B6Cast F_2_ populations). Finally, the biological relevance of the candidate gene with respect to trait was considered.

### Quantification of transcript amounts of candidate genes

For RNA analysis, one male was chosen of 6 to 8 strains carrying the B6 or D2 allele of the target gene, respectively. Total RNA was isolated from liver using the nucleic acid and protein purification Kit (Machery-Nagel, Düren, Germany) and from epididymal adipose tissue as described previously [66]. RNA quality was checked by calculating the A260 nm/280 nm ratio and agarose gel electrophoresis. Complementary DNA was synthesized from 1 μg of RNA using the AccuScript® High Fidelity Reverse Transcriptase (Stratagene, Agilent Technologies, Waldbronn, Germany). Transcript amounts of *Rsad2* and *Colec11* were quantified in epididymal adipose tissue. Quantitative real time PCR was performed on ViiaTM 7 Real-Time PCR System (Applied Biosystems, Darmstadt, Germany). A total reaction volume of 10 μl contained MasterMix Plus for SYBR® Assay (Eurogentec, Cologne, Germany), 10 ng cDNA and 10 μM gene specific primers. Following primers were used: for *Rsad2* AAGCTGAGGAGGTGGTGCAG and GAAAACCTTCCAGCGCACAG, and for *Colec11* TGGACAACCAGGTCACTCAA and AGCCTGTGCCAGGTATGAAG. Triplicates of every samples were measured following an amplification protocol including a 10 minutes activation step at 95°C and 40 cycles à 15 s at 95°C, 20 s at 60°C and 40 s at 72°C. Gene expression was calculated as relative quantity (RQ) using the ΔΔCt method [67]. As an endogeneous control *Rps25* (primers: TCGACAAAGCGACATACGAC and CCACCCTTTGTGTTTCTGGT) was chosen and gene expression was calculated relative to the B6 strain normalized to a value of 1.

## Competing interests

The authors declare that they have no competing interests.

## Authors’ contributions

CN provided BXD mice for experimental studies and contributed to collection of tissue samples. AD collected the tissue samples and designed and carried out the ATR-FTIR study and analysed the data, and performed and interpreted the QTL analysis and wrote the manuscript. PL designed and supervised the ATR-FTIR study and participated in drafting the manuscript. DN contributed to the design of ATR-FTIR study. MKM performed the gene expression measurements. RA and KS contributed to the statistical analysis. GAB conceived, designed and coordinated the study and helped to draft the manuscript. All authors read and approved the final manuscript.

## Supplementary Material

Additional file 1: Table S1 List of protein coding genes in the QTL region on Chr 12. The QTL region between 26 and 30 Mb is associated with relative content of total, saturated and unsaturated fat, collagen, collagen integrity and lipid to protein ratio in epididymal adipose tissue. Click here for file

Additional file 2: Table S2 List of protein coding genes in the QTL on Chr 17. The QTL region between 46 and 50 Mb is associated with relative content of lipid to protein ratio in the liver. Click here for file

Additional file 3: Table S3 Non-synonymous coding variants of candidate genes in humans. a. Rsad2, b. Colec11. Click here for file
