# Supplementary material for: ATR-FTIR spectroscopy reveals genomic loci regulating the tissue response in high fat diet fed BXD recombinant inbred mouse strains
Source: BMC Genomics. 2013 Jun 10;14:386. doi: 10.1186/1471-2164-14-386 (PMC3717084; doi:10.1186/1471-2164-14-386)
Supplement: Additional file 1: Table S1 — List of protein coding genes in the QTL region on Chr 12. The QTL region between 26 and 30 Mb is associated with relative content of total, saturated and unsaturated fat, collagen, collagen integrity and lipid to protein ratio in epididymal adipose tissue. [file 1471-2164-14-386-S1.doc]

**Additional File 1: Table S1. List of protein coding genes in the QTL region on Chr 12.** The QTL region between 26 and 30 Mb is associated with relative content of total, saturated and unsaturated fat, collagen, collagen integrity and lipid to protein ratio in epididymal adipose tissue.

| Symbol | Description | Location (Chr, Mb) |
| --- | --- | --- |
| C920021A13 | 2 days neonate thymus thymic cells cDNA, RIKEN full-length enriched library, clone:C920021A13 product:unknown EST, full insert sequence. | Chr12: 26.071106 |
| A630075K04Rik | RIKEN cDNA A630075K04 (similar to putative histidine kinase) | Chr12: 26.347497 |
| Rnf144 | ring finger protein 144 | Chr12: 26.985873 |
| 4930549C15Rik | RIKEN cDNA 4930549C15 gene | Chr12: 27.056029 |
|  |  |  |
| Rsad2 | radical S-adenosyl methionine domain containing 2 | Chr12: 27.127800 |
| Tyki | thymidylate kinase family LPS-inducible member | Chr12: 27.161892 |
| 4930435E18Rik | RIKEN cDNA 4930435E18 gene | Chr12: 27.499509 |
| 9830137M10Rik | RIKEN cDNA 9830137M10 gene | Chr12: 27.514510 |
| C030020L09Rik | ESTs | Chr12: 27.819066 |
| AI604832 | adult retina cDNA, RIKEN full-length enriched library, clone:A930017G19 product:hypothetical protein, full insert sequence. | Chr12: 27.825848 |
| Sox11 | SRY-box 11; far 3' UTR | Chr12: 28.019523 |
| 1110015M06Rik | RIKEN cDNA 1110015M06 gene | Chr12: 29.122714 |
| 1700022F17Rik | RIKEN cDNA 1700022F17 gene | Chr12: 29.154778 |
| Allc | allantoicase | Chr12: 29.238675 |
| Colec11 | collectin sub-family member 11 | Chr12: 29.279091 |
| Rps7 | ribosomal protein S7 | Chr12: 29.316015 |
| Rnaseh1 | ribonuclease H1; mid to distal 3' UTR | Chr12: 29.344077 |
| Adi1 | acireductone dioxygenase 1; mid 3' UTR | Chr12: 29.366465 |
| Ttc15 | tetratricopeptide repeat domain 15 | Chr12: 29.375914 |
| 1110006E14Rik | ESTs | Chr12: 29.409481 |
| Ttc15 | tetratricopeptide repeat domain 15 | Chr12: 29.420401 |
| A630089K24Rik | 3 days neonate thymus cDNA, RIKEN full-length enriched library, clone:A630090A15 product:unknown EST, full insert sequence. | Chr12: 29.634421 |
| 4833405L11Rik | RIKEN cDNA 4833405L11 gene | Chr12: 29.789095 |
