# Supplementary material for: ATR-FTIR spectroscopy reveals genomic loci regulating the tissue response in high fat diet fed BXD recombinant inbred mouse strains
Source: BMC Genomics. 2013 Jun 10;14:386. doi: 10.1186/1471-2164-14-386 (PMC3717084; doi:10.1186/1471-2164-14-386)
Supplement: Additional file 2: Table S2 — List of protein coding genes in the QTL on Chr 17. The QTL region between 46 and 50 Mb is associated with relative content of lipid to protein ratio in the liver. [file 1471-2164-14-386-S2.doc]

**Additional File 2: Table S2. List of protein coding genes in the QTL on Chr 17.** The QTL region between 46 and 50 Mb is associated with relative content lipid to protein ratio in the liver.

| Symbol | Description | Location (Chr, Mb) |
| --- | --- | --- |
| Vegfa | vascular endothelial growth factor A; mid-distal 3' UTR | Chr17: 46.154213 |
| AU040583 | AU040583 EST; well expressed sequence (putative non-coding) | Chr17: 46.233479 |
| Mrps18a | mitochondrial ribosomal protein S18A | Chr17: 46.248708 |
| 1700027N10Rik | RIKEN cDNA 1700027N10 gene | Chr17: 46.271921 |
| Mad2l1bp | mitotic arrest deficient 2, homolog-like 1 (MAD2L1) binding protein; last exon and 3' UTR | Chr17: 46.284624 |
| Gtpbp2 | GTP binding protein 2 | Chr17: 46.298683 |
| Polh | polymerase (DNA directed), eta (RAD 30 related) | Chr17: 46.308369 |
| Xpo5 | exportin 5 (pre-miRNA and dicer transport); last four exons | Chr17: 46.377762 |
| Rpo1-1 | RNA polymerase 1-1 | Chr17: 46.380921 |
| D17Wsu94e | DNA segment, Chr 17, Wayne State University 94, expressed | Chr17: 46.388408 |
| Gm88 | Gene model 88, (NCBI); putative exon (from EST AK134769) | Chr17: 46.393989 |
| Tjp4 | tight junction protein 4 (peripheral) | Chr17: 46.394957 |
| Egfl9 | EGF-like-domain, multiple 9 | Chr17: 46.439745 |
| Dlk2 | delta-like 2 homolog; last exon and proximal to mid 3' UTR | Chr17: 46.439886 |
| Abcc10 | ATP-binding cassette, sub-family C (CFTR/MRP), member 10; last four exons | Chr17: 46.440563 |
| Zfp318 | zinc finger protein 318; exons 3, 4, and 5 (transQTL on chr 4 in BXD Eye Data) | Chr17: 46.536814 |
| G1p2 | interferon, alpha-inducible protein | Chr17: 46.543907 |
| Zfp318 | zinc finger protein 318 | Chr17: 46.557374 |
| Crip3 | cysteine-rich protein 3 | Chr17: 46.567949 |
| Slc22a7 | solute carrier family 22 (organic anion transporter), member 7; second and third to last exons and proximal to mid 3' UTR | Chr17: 46.569307 |
| C330008L01Rik | RIKEN cDNA C330008L01 gene | Chr17: 46.578785 |
| Ttbk1 | tau tubulin kinase 1; 3' UTR | Chr17: 46.579442 |
| 4931408A02Rik | RIKEN cDNA 4931408A02 gene | Chr17: 46.591116 |
| BC048355 | cDNA sequence BC048355 | Chr17: 46.636033 |
| Parc | p53-associated parkin-like cytoplasmic protein | Chr17: 46.637715 |
| Srf | serum response factor serum response factor (smooth muscle differentiation); proximal 3' UTR | Chr17: 46.685391 |
| Ptk7 | PTK7 protein tyrosine kinase 7 | Chr17: 46.701696 |
| Klc4 | kinesin light chain 4; last three exons and 3' UTR | Chr17: 46.767691 |
| 1200014P03Rik | RIKEN cDNA 1200014P03 gene | Chr17: 46.776840 |
| Mrpl2 | mitochondrial ribosomal protein L2; exons 3, 4, 5, and 6 | Chr17: 46.785306 |
| Cul7 | cullin 7 | Chr17: 46.800560 |
| BC011248 | cDNA sequence BC011248; last exon and 3' UTR (strong transQTL on chr 4 in BXD eye data) | Chr17: 46.804614 |
| Klhdc3 | kelch domain containing 3; 3' UTR | Chr17: 46.811502 |
| Mea1 | male enhanced antigen 1; first exon | Chr17: 46.818623 |
| Ppp2r5d | protein phosphatase 2, regulatory subunit B (B56), delta isoform | Chr17: 46.820101 |
| Pex6 | peroxisomal biogenesis factor 6; first exon | Chr17: 46.849248 |
| Gnmt | glycine N-methyltransferase | Chr17: 46.862736 |
| Cnpy3 | canopy 3 (trinucleotide repeat containing 5); proximal half of 3' UTR | Chr17: 46.873069 |
| Ptcra | pre T-cell antigen receptor alpha | Chr17: 46.892915 |
| 2310039H08Rik | RIKEN cDNA 2310039H08 gene | Chr17: 46.909780 |
| Rpl7l1 | ribosomal protein L7-like 1 | Chr17: 46.911040 |
| BC032203 | cDNA sequence BC032203; far 3' UTR | Chr17: 46.935290 |
| KIAA0240 | hypothetical protein KIAA0240 | Chr17: 46.937913 |
| 2600002F22Rik | ESTs, Weakly similar to RIKEN cDNA 5730493B19 [] [M.musculus] | Chr17: 46.990842 |
| A230078I05Rik | A230078I05Rik CNS midbrain EST | Chr17: 46.993195 |
| BE995645 | ESTs, Weakly similar to Ser/Arg-related nuclear matrix protein; plenty-of-prolines-101; serine/arginine repetitive matrix protein 1 [] [M.musculus] | Chr17: 46.993782 |
| 5630400M01Rik | ESTs | Chr17: 47.008879 |
| Tbcc | tubulin-specific chaperone c | Chr17: 47.028243 |
| Prhp2 | peripherin 2 (retinal degeneration slow, retinitis pigmentosa 7 tetraspanin protein); mid distal 3' UTR | Chr17: 47.061287 |
| Ubr2 | ubiquitin protein ligase E3 component n-recognin 2 | Chr17: 47.064959 |
| E130209G04Rik | RIKEN cDNA E130209G04 gene | Chr17: 47.147601 |
| Trerf1 | transcriptional regulating factor 1 | Chr17: 47.490012 |
| Mrps10 | mitochondrial ribosomal protein S10 | Chr17: 47.507089 |
| Guca1b | guanylate cyclase activator 1B (photoreceptor-restricted calcium-binding guanylin 2, retinitis pigmentosa); intron 1 (possible short form 3' UTR) | Chr17: 47.523753 |
| Guca1a | guanylate cyclase activator 1a (cone dystrophy 3); exons 1, 2, and 3 | Chr17: 47.532503 |
| 1700001C19Rik | RIKEN cDNA 1700001C19 gene | Chr17: 47.550387 |
| LOC224833 | similar to bM573K1.5 (novel Ulp1 protease family member) | Chr17: 47.599806 |
| Tbn | taube nuss | Chr17: 47.625518 |
| Wdr5b | WD repeat domain 5B | Chr17: 47.656287 |
| 5830436I19Rik | RIKEN cDNA 5830436I19 gene | Chr17: 47.667403 |
| 4732474A20Rik | ESTs | Chr17: 47.681807 |
| A030011A13Rik | BB167641 EST (Foxp4 or Ccnd3 associated); intron of Foxp4 | Chr17: 47.686932 |
| 4933417N07Rik | ESTs | Chr17: 47.692368 |
| 9230106B05Rik | RIKEN cDNA 9230106B05 gene | Chr17: 47.715727 |
| Ccnd3 | cyclin D3 | Chr17: 47.732286 |
| Bysl | bystin-like; mid 3' UTR | Chr17: 47.737363 |
| Usp49 | ubiquitin specific protease 49 | Chr17: 47.760610 |
| Mia2 | adult male urinary bladder cDNA, RIKEN full-length enriched library, clone:9530064D18 product:unknown EST, full insert sequence. | Chr17: 47.765185 |
| C330046L10Rik | ES cells cDNA, RIKEN full-length enriched library, clone:C330046L10 product:hypothetical Ubiquitin carboxyl-terminal hydrolase family 2 containing protein, full insert sequence. | Chr17: 47.809188 |
| Tomm6 | translocase of outer mitochondrial membrane 6; distal 3' UTR | Chr17: 47.823697 |
| 4930417B13Rik | expressed sequence AI449674 | Chr17: 47.836938 |
| Frs3 | fibroblast growth factor receptor substrate 3 | Chr17: 47.840738 |
| Pgc | progastricsin (pepsinogen C); last 4 exons (transQTL on chr 4 in BXD Hippocampus Data) | Chr17: 47.870703 |
| Tcfeb | transcription factor EB; proximal to distal 3 UTR | Chr17: 47.928844 |
| Mdfi | MyoD family inhibitor | Chr17: 47.952399 |
| Foxp4 | forkhead box P4 | Chr17: 48.004101 |
| 1700122O11Rik | RIKEN cDNA 1700122O11 gene | Chr17: 48.173800 |
| Trem1 | triggering receptor expressed on myeloid cells 1 | Chr17: 48.380650 |
| Trem3 | triggering receptor expressed on myeloid cells 3 | Chr17: 48.389007 |
| Treml4 | triggering receptor expressed on myeloid cells-like 4 | Chr17: 48.414134 |
| AW049306 | AV140744 | Chr17: 48.439724 |
| C030013G03Rik | RIKEN cDNA C030013G03 gene | Chr17: 48.449968 |
| AW049306 | ESTs | Chr17: 48.451290 |
| B430306N03Rik | RIKEN cDNA B430306N03 gene | Chr17: 48.464296 |
| Trem2 | triggering receptor expressed on myeloid cells 2 (Alzheimer's disease associated) | Chr17: 48.487839 |
| Treml1 | triggering receptor expressed on myeloid cells-like 1 | Chr17: 48.505340 |
| Nfya | nuclear transcription factor-Y alpha | Chr17: 48.526326 |
| AI314976 | expressed sequence AI314976 | Chr17: 48.556089 |
| Apobec2 | apolipoprotein B editing complex 2 | Chr17: 48.558660 |
| Apobec2 | apolipoprotein B editing complex 2; antisense in 3' UTR or last intron | Chr17: 48.559639 |
| Bzrpl1 | benzodiazapine receptor, peripheral-like 1 | Chr17: 48.588068 |
| Unc5cl | unc-5 homolog C (C. elegans)-like | Chr17: 48.607516 |
| Lrfn2 | leucine rich repeat and fibronectin type III domain containing 2 | Chr17: 49.236323 |
| 1700008K24Rik | RIKEN cDNA 1700008K24 gene | Chr17: 49.251668 |
| Mocs1 | molybdenum cofactor synthesis 1 | Chr17: 49.567865 |
| Abhd3 | polymorphic long terminal repeat; LTR maps to chr 17 | Chr17: 49.586532 |
| Daam2 | dishevelled associated activator of morphogenesis 2; distal 3' UTR | Chr17: 49.595409 |
| Kif6 | kinesin family member 6 | Chr17: 49.894431 |
| 4930556A20Rik | RIKEN cDNA 4930556A20 gene | Chr17: 49.959482 |
