# Supplementary material for: ATR-FTIR spectroscopy reveals genomic loci regulating the tissue response in high fat diet fed BXD recombinant inbred mouse strains
Source: BMC Genomics. 2013 Jun 10;14:386. doi: 10.1186/1471-2164-14-386 (PMC3717084; doi:10.1186/1471-2164-14-386)
Supplement: Additional file 3: Table S3 — Non-synonymous coding variants of candidate genes in humans. a. Rsad2, b. Colec11. [file 1471-2164-14-386-S3.doc]

| Marker ID | Chr: bp | Alleles | Class | Type | Amino Acid | AA co-ordinate | SIFT | PolyPhen | Transcript |
| --- | --- | --- | --- | --- | --- | --- | --- | --- | --- |
| rs35430708 | 2:7017951 | C/A | SNP | Non-synonymous coding | A/D | 7 (2) | **deleterious** | benign | ENST00000382040 |
| rs111568894 | 2:7018011 | G/A | SNP | Non-synonymous coding | S/N | 27 (2) | **deleterious** | benign | ENST00000382040 |
| rs17851586 | 2:7018056 | T/G | SNP | Non-synonymous coding | L/R | 42 (2) | tolerated | benign | ENST00000382040 |
| rs112087451 | 2:7018074 | A/G | SNP | Non-synonymous coding | K/R | 48 (2) | tolerated | benign | ENST00000382040 |
| rs2305257 | 2:7018085 | G/A | SNP | Non-synonymous coding | V/I | 52 (1) | tolerated | benign | ENST00000382040 |
| rs61740841 | 2:7027258 | C/T | SNP | Non-synonymous coding | T/M | 234 (2) | tolerated | **possibly damaging** | ENST00000382040 |
| rs76802786 | 2:7019157 | T/C | SNP | Non-synonymous coding | S/P | 4 (1) | tolerated | **deleterious** | ENST00000541728 |
| rs61740841 | 2:7027258 | C/T | SNP | Non-synonymous coding | T/M | 127 (2) | **deleterious** | benign | ENST00000541728 |

**Additional File 3: Table S3**. **Non-synonymous coding variants of candidate genes in humans.**

**a.** *Rsad2*

**Table S3 continued**

**b. *Colec11***

| Marker ID | Chr: bp | Alleles | Class | Type | Amino Acid | AA co-ordinate | SIFT | PolyPhen | Transcript |
| --- | --- | --- | --- | --- | --- | --- | --- | --- | --- |
| rs113532503 | 2:3653800 | A/T | SNP | Non-synonymous coding | S/C | 7 (1) | tolerated | benign | ENST00000402794 |
| rs113532503 | 2:3653800 | A/T | SNP | Non-synonymous coding | S/C | 7 (1) | tolerated | benign | ENST00000402922 |
| rs113532503 | 2:3653800 | A/T | SNP | Non-synonymous coding | S/C | 7 (1) | tolerated | **possibly damaging** | ENST00000403096 |
| rs113532503 | 2:3653800 | A/T | SNP | Non-synonymous coding | S/C | 7 (1) | tolerated | benign | ENST00000404205 |
| rs112639715 | 2:3653813 | G/C | SNP | Non-synonymous coding | C/S | 11 (2) | **deleterious** | benign | ENST00000402794 |
| rs112639715 | 2:3653813 | G/C | SNP | Non-synonymous coding | C/S | 11 (2) | tolerated | benign | ENST00000402922 |
| rs112639715 | 2:3653813 | G/C | SNP | Non-synonymous coding | C/S | 11 (2) | tolerated | benign | ENST00000403096 |
| rs112639715 | 2:3653813 | G/C | SNP | Non-synonymous coding | C/S | 11 (2) | tolerated | benign | ENST00000404205 |
| rs62107197 | 2:3660935 | C/T | SNP | Non-synonymous coding | P/L | 26 (2) | - | unknown | ENST00000236693 |
| rs112274120 | 2:3673680 | A/G | SNP | Non-synonymous coding, Splice site | Q/R | 64 (2) | **deleterious** | benign | ENST00000236693 |
| rs113757131 | 2:3691448 | A/G | SNP | Non-synonymous coding | N/D | 183 (1) | tolerated | **probably damaging** | ENST00000236693 |
| rs113757131 | 2:3691448 | A/G | SNP | Non-synonymous coding | N/D | 186 (1) | **deleterious** | **probably damaging** | ENST00000349077 |
| rs113757131 | 2:3691448 | A/G | SNP | Non-synonymous coding | N/D | 162 (1) | **deleterious** | **probably damaging** | ENST00000382062 |
| rs113757131 | 2:3691448 | A/G | SNP | Non-synonymous coding | N/D | 136 (1) | tolerated | **probably damaging** | ENST00000402794 |
| rs113757131 | 2:3691448 | A/G | SNP | Non-synonymous coding | N/D | 136 (1) | **deleterious** | **probably damaging** | ENST00000402922 |
| rs113757131 | 2:3691448 | A/G | SNP | Non-synonymous coding | N/D | 160 (1) | tolerated | **probably damaging** | ENST00000403096 |
| rs113757131 | 2:3691448 | A/G | SNP | Non-synonymous coding | N/D | 112 (1) | tolerated | **probably damaging** | ENST00000404205 |
| rs113757131 | 2:3691448 | A/G | SNP | Non-synonymous coding | N/D | 200 (1) | **deleterious** | **probably damaging** | ENST00000418971 |
| rs7567833 | 2:3691548 | A/G | SNP | Non-synonymous coding | H/R | 216 (2) | tolerated | benign | ENST00000236693 |
| rs7567833 | 2:3691548 | A/G | SNP | Non-synonymous coding | H/R | 219 (2) | tolerated | benign | ENST00000349077 |
| rs7567833 | 2:3691548 | A/G | SNP | Non-synonymous coding | H/R | 195 (2) | tolerated | benign | ENST00000382062 |
| rs7567833 | 2:3691548 | A/G | SNP | Non-synonymous coding | H/R | 169 (2) | tolerated | benign | ENST00000402794 |
| rs7567833 | 2:3691548 | A/G | SNP | Non-synonymous coding | H/R | 169 (2) | tolerated | benign | ENST00000402922 |
| rs7567833 | 2:3691548 | A/G | SNP | Non-synonymous coding | H/R | 193 (2) | tolerated | benign | ENST00000403096 |
| rs7567833 | 2:3691548 | A/G | SNP | Non-synonymous coding | H/R | 145 (2) | tolerated | benign | ENST00000404205 |
| rs7567833 | 2:3691548 | A/G | SNP | Non-synonymous coding | H/R | 233 (2) | tolerated | benign | ENST00000418971 |
